# The Therapist as Conditioned Stimulus

**DOI:** 10.5334/pb.454

**Published:** 2018-07-26

**Authors:** Paul Eelen, Eric Depreeuw, Omer Van Den Bergh

**Affiliations:** 1 Faculty of Psychology and Educational Sciences, KU Leuven, BE

**Keywords:** therapeutic relationship, empathy, learning principles

## Abstract

This manuscript is part of a special issue to commemorate professor Paul Eelen, who passed away on August 21, 2016. Paul was a clinically oriented scientist, for whom learning principles (Pavlovian or operant) were more than salivary responses and lever presses. His expertise in learning psychology and his enthusiasm to translate this knowledge to clinical practice inspired many inside and outside academia. Several of his original writings were in the Dutch language. Instead of editing a special issue with contributions of colleagues and friends, we decided to translate a selection of his manuscripts to English to allow wide access to his original insights and opinions. Even though the manuscripts were written more than two decades ago, their content is surprisingly contemporary. This manuscript was originally published in 1989 as part of an edited book on the therapeutic relation. In this chapter, Paul Eelen takes a critical position against the dominance of the client-centered approach. He presents the therapeutic relation as a behavioural interaction between the patient and his therapist which is subject to laws of learning. This is exemplified by an in-depth analysis of the therapist as a conditioned stimulus.

First published as: Eelen, P., Depreeuw, E., & Van den Bergh, O. (1989). De therapeut als geconditioneerde stimulus. In H. Vertommen, G. Cluckers, & G. Lietaer (Eds.), *De relatie in therapie* (pp. 147–165). Leuven: Universitaire Pers Leuven.^1^

Although these are times of therapeutic ecumenism, it is appropriate to reflect, from time to time, on what distinguishes existing therapeutic methods. A reflection on the contrast between behaviour therapy and client-centered therapy seems in order, given the context in which the idea for this book was shaped. The therapist-client (T-C) relationship, the subject of this book, offers a great departure point for such an exercise.^2^ Hence the slightly provocative title! To label the therapist a conditioned stimulus at first glance appears to be fairly at odds with the conceptual framework in which the T-C relationship is described from a client-centered therapy perspective. This aptly summarises the two-part focus of this contribution. The first paragraph explains why behaviour therapists remain slightly hesitant to accept the basic philosophy underlying client-centered therapy. The second paragraph discusses how the T-C relationship can be situated in a conceptual framework from a behaviour therapy perspective.

## The genuine, unconditional and emphatic therapist

Naturally, the critical observations contained in this paragraph might be focused on a “straw man” rather than on what really happens in client-centered therapy. We know from experience that statements about behaviour therapy made by other disciplines, often seem unrecognisable. We, in turn, run that same risk in this outsider position. We take consolation in the fact that “the insiders” will know better!

Still, these critical observations seem justified because, all too often, the opinion is expressed that client-centered therapy includes the so-called “core conditions” that every therapist, no matter what school, should have received training in. True ecumenism, then, appears to lie in the teaching of relational skills through client-centered therapy and “technical expertise” through behaviour therapy. This reasoning is even somewhat the basis for the training programme for clinical psychologists in Leuven today.

As far as we are concerned, this is a misplaced ecumenical movement because the differences between the approaches are still too large to treat them as one. That, at least, is the impression we have after having browsed the literature on client-centered therapy. What is striking from our reading of this literature is (a) the poor operationalisation of the key concepts, which probably stems from matters of principle rather than technical reasons; (b) the limited reference to more general psychological concepts; and (c) that the concepts of “experience” and “behaviour” have been disconnected. Each of these observations is discussed below, taking into account the abovementioned outsider position.

First, there is the *poor operationalisation of the central concepts*, particularly with respect to the therapist’s so-called “core conditions”.

Carl Rogers must have been in a very optimistic state of mind when, in 1957, he published an article with the promising title *The Necessary and Sufficient Conditions of Therapeutic Personality Change*. On eight pages, a summary is offered of the necessary and sufficient conditions that researchers and therapists had been searching for since many years (and, Rogers’ article notwithstanding, are still fruitlessly searching for to this date).

Six conditions are necessary in Roger’s view: (a) there needs to be a psychological *relationship* between two persons, (b) with one of these two (the client) experiencing a state of *incongruence*, and the other (the therapist) demonstrating the core conditions of (c) *genuineness* (congruence), (d) *unconditional positive regard* and (e) *empathy*, that of course (f) also have to be *perceived* by the client as such. When these conditions are met during therapy, they are also sufficient to bring about a change process in the client.

Rogers was surprised by the “simplicity of what has emerged” (p. 45); he was even more surprised when he realised that, at first glance, so many elements were not included as necessary and sufficient that were always mentioned as important in the then existing literature. These conditions are considered necessary and sufficient (a) irrespective of the client’s problem, (b) irrespective of the therapeutic orientation one adheres to, (c) irrespective of the therapeutic setting, (d) irrespective of one’s professional training and (e) with no need of assessment.

The author’s name, the chosen title and its presentation as a series of hypotheses made this article a milestone in the evolution of client-centered therapy. The author’s name guaranteed that its contents were based on a contemplation on years of therapeutic practice. Had the same article been written by a novice therapist, it would have barely been considered suitable for publication and it certainly would not have had the same influence. The title held quite a few scientific promises. Every empirical science after all aims to explain a phenomenon (in this case, the change in the client) by describing its sufficient and ideally also its necessary conditions. Using logic notation from philosophy of science, this is represented as: if *p* then *q* and if *q* then *p*, or *p* ⇔ *q*. But this presupposes that both p and q can be objectified in an unambiguous fashion.

And this is where the trouble lies. Rogers himself admits in the article that such an operationalisation of both the conditions (p) and the change process in the client (q) is largely absent today. Still in the same optimistic mood, he nonetheless offers a design for what such measurements might look like, even though his suggestions remain limited to the listing of items in a questionnaire.

More sophisticated measurement instruments today exist to assess Rogers’ statements. Irrespective of the result of this research, the operationalisation of these necessary and sufficient conditions nevertheless remains problematic – due to principal more than technical reasons. On the one hand, painstaking attempts are made to avoid translating these conditions into objective criteria. Whereas Rogers’ initial emphasis (1951) was on the technical aspects (what should you *do* so that empathy, genuineness etc. are expressed in the T-C relation), he gradually and increasingly moved away from this: *to be* rather than *to do* is what matters. At stake are the therapist’s internal positions that will (*spontaneously?*) be expressed and externalised as a result, possibly in a very idiosyncratic fashion.

The externalisation, the communication of empathy is not what is most important to me: the inner empathic experience is what is fundamental and it is the source itself for the therapeutic activity. I feel at my best when the expression of my empathy occurs naturally – when it doesn’t require me to think, to ask questions, to purposefully intervene in particular ways. (Rombauts, 1984, p. 173, our translation)

On the other hand, it is assumed that these core conditions nonetheless are objectifiable since external evaluators are assumed to evaluate them through the measuring scales that have been designed for this purpose. It is moreover assumed – and this was one of *the* necessary conditions for Rogers – that the client can perceive these core conditions in the therapist; however, concrete (verbal and non-verbal) behaviour rather than an inner core condition is observed.

In short, what Rogers and many researchers in client-centered therapy later labelled as “therapeutic factors” are not factors but consequences of certain, insufficiently explained factors. To refer back to the abovementioned symbols: they are *q*s of which the *p*s remain unclear. The extensive research that Rogers’ article inspired, remained primarily correlational in nature; it ultimately yielded a poor crop (Lietaer, 1983). One of the reasons for this poor crop, according to Lietaer, is probably the fact that client-centered research was too reductionist. In our opinion, the reason is in fact precisely the opposite: the research and the research question was not reductionist enough. Humanistic psychology in general and client-centered therapy in particular appears to want to avoid every form of reductionism, which threatens to snuff out everything scientific about it. After all, science by definition reduces the overall experience.

We here arrive at an issue that remains characteristic for the further evolution of client-centered therapy, namely the *limited reference to more general psychology literature*. This unjustifiably perpetuates the gap between psycho-therapy and psycho-logy. Rogers views a training in psychology neither as a sufficient nor as necessary condition to the development of a helping relationship. Anyone who is involved in a psychotherapy training programme will subscribe to this view. By analogy, one could state that one does not need pedagogical training to be a good father or mother. But unlike parenthood, psychotherapy concerns the exercise of a *profession* that can and should require a certain level of expertise that exceeds the above-described core conditions and that should have as its foundation a thorough training in what psychology has accrued in scientific knowledge. This knowledge then, is indeed not a sufficient and necessary condition to help a fellow human; it is, however, a necessary condition to be able to position oneself as an expert. It is not just advisable that we have this knowledge at the back of our mind when someone approaches us for professional help; it is *our duty*.

One of Rogers’ greatest accomplishments remains that he called the exclusive expertise of psychiatrists in the practise of psychotherapy into question, and that he demanded the right for clinical psychologists to practise psychotherapy. This should not, however, result in a plea that any person who is empathic, genuine and accepting in their relationship with others should be appointed as therapist. Psychotherapy is applied psychology and it should ideally be practised by psychologists. The often limited psychology training of psychiatrists-psychotherapists is very regrettable in this regard.

It seems to us that client-centered therapy has not sufficiently emphasised this necessity of embedding therapeutic practice in scientific knowledge and that it has, as a result, often provided a breeding ground for training institutes that regard the basic psychology training of participants as of secondary importance. It is for instance notable that Gendlin’s (1981) work on focusing does not make a single reference to the comprehensive psychology theories on emotion and affect. The exceptions to this rule confirm it. There was, for instance, a great deal of affinity between (cognitive) behaviour therapy and Wexler and Rice’s attempt (1974) to describe client-centered therapy within the framework of information-processing theories. This attempt, however, has not always been met with the approval of insiders (De Haas, 1984). A similar affinity exists with Greenberg and Safran’s work (1987) on emotion. As such attempts also start to pave the way for an increasingly mutual conceptual framework and uniform research strategies, a certain level of ecumenical movement appears to become both possible and justified.

Finally, we would like to briefly reflect on what is usually cited as the main difference between client-centered therapy and behaviour therapy. *One approach is supposed to primarily focus its attention on the “experience” of the client, while the other engages with behaviour (with the epithet “merely” usually added to the latter)*. However, the theoretical foundation used to support this contrast appears to be rather weak, unless one continues to unjustifiably identify behaviour therapy with orthodox behaviourism à la Watson.

We are not at all opposed to the claim that what matters in therapy is the client’s lived experience, his “felt experience”. But one does not have direct access to this experience. Continuing to believe in this means returning to classical introspective psychology. The only thing one can do is to trace the requirements, the conditions (independent variables) that influence this “felt experience” (dependent variable), in a way similar to that of a behaviour therapist who does not directly alter the behaviour but suggests changes in the antecedent or consequent factors that determine the behaviour. This *experimental* conceptual model is the core nature of behaviour therapy, which in turn causes it to remain at odds with the fundamental philosophy of client-centered therapy.

## The Therapist as a Conditioned Stimulus

### Technique Versus Relationship?

Behaviour therapy is often criticised for not paying attention to the T-C relationship and for even denying its significance. Such criticisms are partly unfounded. Although behaviour therapy has demystified the importance of the T-C relationship to a certain degree (Walker et al., 1981), it is not a topic that has not at all been discussed (for multiple overviews, see Wilson & Evans, 1977; De Voge & Beck, 1978; Sweet, 1984; Hoorens, 1986).

On the other hand, it cannot be denied that the conceptualisation of this T-C relationship has remained largely absent. The T-C relationship is considered the frame in which the actual procedures for behaviour change, the so-called *techniques*, can be applied. Many even argue that change does not occur inside but outside therapy. At its most extreme, therapy is then reduced to the assigning and correcting of homework. Upon closer investigation, this division between “relationship” and “techniques” appears to be artificial as the technique in many ways is built into the relationship and the other way around. This artificial aspect is present in the (limited) research that has been conducted from a behaviour therapy angle so as to distil the influence of the relationship versus the technique. This research unjustly mirrors itself to the “pills research” that has been conducted in medicine. For instance, attempts have been made to apply the systematic desensitisation (SD) technique by means of a computer and to then compare these results with those of SD applied in a T-C relationship (Lang, Melamed, & Hart, 1970). Disregarding the results of this research for a second allows us to contemplate the question of whether such an approach is even capable of offering clarification regarding the role of the relationship versus the technique. Must we conclude that the relationship is of no importance or that a good relationship with a computer is also possible, if a computer and a therapist do just as good a job?^3^ If the computer very clearly does not do as well – and let us assume this for the sake of our profession – does this then mean that the relationship is an added value to the technique, or that the computer is not sufficiently capable of applying the technique? A study that applied SD through both a “cold” and a “warm” therapist appears to have a similarly fuzzy method in our view (Morris & Suckerman, 1974a, 1974b). The reasoning was the following: the technique is present in both conditions, but the relationship is added only by the warm therapist. If, however, a cold therapist, due to prior experiences, evokes so much distrust, resistance and opposition that he is not listened to, such a study would not be very different from a comparison between SD with instructions in Chinese (potentially even offered in a “warm” fashion) and instructions in one’s native language.

In short, we do not think it is fruitful to view the relationship only as the framework in which the actual therapeutic techniques can be applied. To ask whether a therapeutic effect must be ascribed to a technique or to a relationship is to ask an impossible question as “the relationship” is a too multi-faceted concept to offer a decisive answer.

So how can one then sensibly discuss the relationship from a learning model?

### The T-C relationship and the Operant Learning Model

Several studies were completed in the fifties and sixties of the 20th century from the *operant learning model* under the banner of “verbal conditioning”, with the therapist (or experimenter) functioning as a social reinforcer. The most famous of these are probably the “hm-hm” experiments, which systematically influenced a test subject’s verbal behaviour in terms of contents and form, and this in a more experimental context as well as in the context of a conversation. This was immediately used to demonstrate that such a directive influence was also present in the Rogerian approach that was then still viewed as the most non-directive therapy (see, for instance Rogers, 1960). In hindsight, the research aims and questions of these studies often come across as somewhat naïve. This was often due to the prior definition of a social reinforcer, which was severed from the context in which, the therapist (experimenter) through whom, and the client (test subject) to whom this reinforcer is administered. This was redressed in later studies (for an overview, see De Voge & Beck, 1978). From a Skinnerian point of view – in which a “reinforcer” is always defined in accordance with its effect on the behaviour – such a redressal was but appropriate. It is sometimes forgotten that Skinner was one of the biggest promoters of N = 1 research, or the “person-centered” research.

No matter how limited the focus of these studies was, they succeeded in drawing attention to the fact that a therapist’s verbal and/or non-verbal behaviour has a strong influence on the client’s behaviour. Something that appears to be self-evident at first; yet how often is this influence not underestimated, also outside the context of therapy?

These studies approached the relationship from one viewpoint only: the influence of the therapist (or experimenter) on the client (participant). The research question only becomes truly relational when the interactional element is addressed. Like in the famous cartoon of two rats in the Skinner box, with one rat saying to the other: “Look what a good job I did of conditioning that researcher: when I push the lever, he gives me food!”. Obviously, something similar occurs in therapy: the therapist influences the client, but the latter in turn also influences the therapist. In more recent literature, we can see the gradual development of more established research methods that aim to get a grip on this process of mutual influence. Particularly, methodologies such as sequential analyses are increasingly being applied in the different therapy orientations, although the unit of analysis is often defined in very different ways (Russell & Trull, 1986; Revensdorf et al., 1984; Schindler, 1988). Whether these new methodologies offer a valuable contribution remains to be seen. Still, they appear to offer an important complement to both questionnaires and the experimental research that is often limited to the manipulation of one aspect.

Similarly promising seems the research that was inspired by an ethological approach. Consider, for instance, Bouhuys and Van den Hoofdakker’s study (1986) on depressed patients. Non-verbal aspects of the therapist-client interaction (for instance, eye contact, hand movements, etc.) are carefully observed and recorded. It is shown that a prognosis can already be made regarding a therapy’s successfulness, based on this non-verbal behaviour in the first conversation. This type of study is of course difficult to perform in a normal practice setting. It does, however, make us sensitive to the variables that are otherwise seldom made explicit. And is this not – at the end of the day – the purpose of research?

Both the sequential analysis and the ethological approach do not have a specific behaviour therapeutic focus, but they are compatible with it because they fully subscribe to a functionally experimental conceptual framework.

No matter how mutual the influence between T and C, in our opinion, the therapist will have to direct this process. To again use the example of the rat cartoon: the rats might think that they have conditioned the test leader, but the latter knows better! Directing here means knowing which way to go, formulating an objective. This is precisely where behaviour therapy does not fall short. Explicitly discussing the objective with the client does not imply that the means of arriving at that objective are fully made explicit. Just as an experimenter does not fully inform his participant about the study’s independent variable, a therapist similarly should not make his method all too transparent. This of course constitutes a sort of manipulation – that most feared and pejorative term among therapists – but the question is whether we can eventually escape from it. Ever since Skinner’s and Rogers’ historic debate, this theme has often resurfaced in the literature and much of the criticism directed at behaviour therapy is tied to a misunderstanding of the notions of control and manipulation. What to think of a client-centered therapist who tells his client in advance: “Look, I’m going to reflect on your experience every now and then in the hopes that this will help you get deeper into it!” Or consider an analyst who justifies his impersonal behaviour toward the client by hoping that it will cause the client to arrive at a good transfer!

We are manipulators after all! The analyst and the behaviour therapist do this explicitly; the client-centered therapists deny it, but they do it too. As soon as a therapist starts justifying his method either to himself or to his supervisors with “I’m doing this to …” (and he hopefully has such justification), he becomes a manipulator, a practitioner.

### The T-C Relationship and the Pavlovian Learning Model

The conceptual framework of the operant learning paradigm does not suffice to describe the core of a T-C relationship. It does not, after all, in itself offer an answer to the question of why precisely a therapist can become an important social reinforcer or, more generally, why the therapist comes to obtain special significance to the client.

What, in many cases, makes him an emotionally charged figure for the client? Behaviour therapy does not make the answer to this question sufficiently explicit. Wolpe, one of its founding fathers, nevertheless offered a first hint by describing the therapist as an “inhibitory stimulus”. This leads us to the terminology of classical or Pavlovian conditioning. It scarcely deserves mention that this learning paradigm, possibly even more so than the operant model, has fallen victim to caricatural depictions that are true travesties. Even behaviour therapists today comment on it with scorn, especially now that cognitive behaviour therapy has offered them a more modern jargon. This chapter of course is not the place to redress these caricatural depictions (for a more elaborate discussion, see Eelen et al., 1988). Still, it is useful to first shed light on the essence of this paradigm before applying it to the T-C relationship.

### A number of key thoughts on classical conditioning

Classical conditioning is a process through which stimuli (CS; conditioned stimuli) (persons, situations, events) acquire a new meaning because they are contingent on what happens to a stimulus (UCS; unconditioned stimulus) (person, situation, event) that already has a well-defined meaning.^4^

For the purpose of this discussion, we limit this clearly defined meaning to the affective, emotional dimensions of these stimuli, which we roughly define into two categories – “pleasant” and “unpleasant”.

What now can happen to these stimuli? We distinguish between three operations: (a) a meaningful stimulus can be administered; (b) this stimulus can be removed; (c) this stimulus does not occur, even though it is expected. A learning process, in other words, always precedes the latter operation: *nothing* only becomes *something* when a certain expectation has been created in advance.

The UCS nature (pleasant – unpleasant) and the three types of operations can be combined into six possible operations.

In a “pure” classical conditioning procedure, these operations are response-independent and usually involve stimulus manipulations by the experimenter (or by “Mother Nature”). In the operant learning paradigm, these operations can be viewed as the *outcome*, the result of a behaviour in the operant learning paradigm.

Classical conditioning at its essence simply means that (neutral) stimuli are contingent on one of the six possible operations, which causes them to acquire a different meaning. We can also generally describe this new meaning as “pleasant” or “unpleasant”.

It is, however, possible to further slightly differentiate this dichotomous categorisation of pleasant-unpleasant by ascribing a particular affective and emotional meaning to each of these operations (see, for instance: Mowrer, 1960; Gray, 1975, 1987; Bakker-De Pree, 1987). The absence of a positive stimulus, for instance, evokes *frustration* (anger), the administration of a negative stimulus *fear*, and the removal of a positive stimulus *disappointment*. These three affective states can summarily be described as “negative feelings”.

When a negative stimulus fails to appear, *safety* is provided; the administration of a positive stimulus provides *hope* and *joy*; the removal of a negative stimulus *relief*. This produces three separate types of “positive feelings”.^5^

It is remarkable then that classical conditioning has almost always been identified with the change in meaning associated with neutral stimuli that are contingent on the *administration* of a UCS. Familiar prototypes include “Pavlov’s dog”: an auditory or visual stimulus that is followed by food receives a positive valence, while a neutral stimulus that is followed by an aversive stimulus acquires a negative valence. The procedures of what Pavlov dubbed *excitatory conditioning* are sufficiently well-known.

The other operations indicating *inhibitory conditioning*, however, are just as important (and they were also to Pavlov). The absence of an expected stimulus, especially, has grievously been overlooked. For the purposes of clarification, we offer a summary description of the prototypical procedure developed by Pavlov himself to realise conditioned inhibition. Consider, for instance, a situation in which stimulus A is always followed by a UCS. Stimulus A thus gradually induces the expectation that the UCS will be administered (excitatory conditioning). Every now and again, A is administered together with a different neutral stimulus B, and A + B is not followed by the UCS. Under these circumstances, B becomes an inhibitory stimulus. The absence of the UCS administration is as it were ascribed to stimulus B, which causes B to acquire a meaning that is opposite to that of stimulus A. If stimulus A for instance induces fear, stimulus B acquires a meaning of safety.

Although it would be impossible to shed light on all the different facets of inhibitory conditioning in the context of this paper, we do briefly want to note inhibitory conditioning’s role in *extinction*. Let us again further clarify this using a schematic presentation. In a first phase, stimulus A is always followed by the UCS. After this excitatory conditioning, A is presented independently, with the UCS no longer succeeding it. After a while, A again becomes a neutral stimulus (extinction). Let us now assume that stimulus A is presented together with B, a neutral stimulus, from the extinction phase onward. A + B of course are no longer followed by the UCS. Under these conditions, B becomes an inhibitory stimulus, and something highly remarkable occurs: extinction at first glance appears to occur when A + B are offered. This extinction, however, is due to the fact that B partially inhibits the effects of A. As long as both are presented together, extinction appears to be complete. As soon as A is no longer accompanied by B, however, the original conditioning effect is again foregrounded. Recent studies have revealed that the role of stimulus B can also be assumed by context stimuli. This phenomenon possibly plays an important role in addiction, for which the role of conditioning is increasingly becoming the central focus. Further below we will also discuss the related possibility of therapist-addiction.

Before we conclude this discussion of the core concepts associated with classical conditioning and relate it to the T-C relationship, we wish to offer a brief justification for why we believe these conditioning phenomena are relevant for general clinical practice. In the previous pages, we have often used terms as “meaning” and “expectation” when such terms at first glance appear to contrast with the language traditionally used to discuss conditioning. What makes conditioning fascinating, then, is that such terms developed as theoretical constructs from meticulous observations of the behaviour of laboratory animals, whereas clinical practice (as well as behaviour therapy) all too often has a tendency to equate these theoretical constructs with that which a client verbally reports from his phenomenal experience. The danger exists that behaviour therapists as well will overlook the richness of the conditioning model, due to the mushrooming of pseudo-cognitive models.

### Application on the T-C relationship

Wolpe’s abovementioned hint, which described the therapist as an inhibitory stimulus, can serve as a point of departure for application of this conceptual framework to the T-C relation. Inhibitory conditioning occurs when a UCS is removed or remains absent. In case of a positive UCS, the neutral contingent stimulus acquires a “negative” meaning; in case of a negative UCS, the stimulus that is contingent on its removal or ‘remaining absent’ acquires a “positive” meaning.

Particularly in a therapeutic setting, these operations can frequently occur. It is consequently not surprising that the therapist becomes a very ambivalent figure. On the one hand, he is often likely to be the source of the removal or absence of a positive stimulus: a client who expects his therapist to show a great level of intimacy will be inclined to interpret his therapist’s neutral benevolence as dismissive. Feelings of resistance and frustration will develop that may even cause him to end the therapy.

On the other hand, the therapist is often the source of the removal or absence of negative stimuli. Several examples can further illustrate this.

First and foremost, the initial hesitancy to seek a therapist is still considerably strong in the culture we live in. Most clients have already gone through arduous ordeals before they can be persuaded to seek therapy. They have, in the meantime, developed an expectation pattern for the dismissive manner in which their environment responds to their problems and this has caused them to accumulate many frustrations. While the client expects criticism and rejection, both are normally absent with a good therapist.

In addition, it is often the case that a client begins a session in a very negative mood and feels somewhat relieved at the end. This relief – rightly or wrongly – is ascribed to the therapist.

Finally – and this is probably typical for what happens in behaviour therapy – the client often lives in the expectation that he can no longer handle certain things. A client for instance has come to expect that he will always have a panic attack whenever he visits a supermarket. Together with the therapist, he is confronted with the situation that he is fearful of (exposure) and there is no panic attack! At first sight, this appears to be a successful intervention. But there is a considerable danger that the absence of panic is fully ascribed to the therapist’s presence. (This, in fact, is yet another demonstration of the impossibility of separating relations and techniques!) The therapist becomes the *safety signal*, which the client no longer lets go off.

A similar situation occurs when the therapist confronts the client with imaginary stimuli that elicit heavily charged emotions in the client. Think, for instance, of bereavement therapy. At first glance, such an approach appears to be the ideal situation to make the therapist an aversive stimulus. It is precisely this aspect of the procedure, however, that is discussed with the client in advance, so that the likelihood that the experiencing of negative emotions is ascribed to the therapist is small. The conditioning effect of the subsequent emotional relief, which the therapist is of course most associated with, often is not discussed in advance.

These are just a couple of examples to illustrate how the therapist can become an inhibitory stimulus. We would like to relate these examples to a couple of more general considerations.

First, these examples perhaps illustrate that conditioning is not a process that begins from a clean sheet. The conditioning process is determined by a client’s entire prior history and of course that of the therapist as well: the client after all often entertains explicit as well as implicit expectations vis-à-vis the therapist, who in his turn explicitly or implicitly responds to these expectations. No matter what the therapist does, his behaviour and his person cannot remain neutral. From a conditioning perspective, it can even be predicted that such inhibitory conditioning will occur at its strongest in psycho-analytic settings. Given the analyst’s neutral, impersonal attitude, it is not surprising that the therapeutic event in the analysis is almost exclusively situated in this relational field; by behaving meaning-less, the analyst becomes a Rorschach packed with meanings, from resistance to transfer. It is not clear to us whether psychoanalysis also takes into consideration the inhibitory conditioning that can be viewed as more or less as an opposite transfer.^6^

Inhibitory conditioning is what makes the T-C relationship so special – at least to the clients who enter therapy for a specific problem. The therapist to them is often a *person* who offers them safety, comfort. The “healing” aspect of this process cannot be denied. Still, precisely this has a treacherous effect that can deter rather than promote the client’s independence. For the therapist, it is after all very reinforcing at first to experience being that person who offers the client safety and comfort. The therapist interprets what almost seems like a crush on the part of the client as rapprochement behaviour, when it is in fact avoidance behaviour. This is how a therapy addiction – or therapist addiction – develops.

Conditioning literature has shown us that an inhibitory stimulus only extinguishes when there is nothing left to inhibit. And this is where – from a conditioning perspective – the therapist’s almost paradoxical task lies. On the one hand, he is worried about obtaining an extinction of feelings of fear, aggression or blame by “using techniques”. Because he is himself a central stimulus in this extinction process, all the requirements for inhibitory conditioning towards his person are met. This in fact causes him to hamper the extinction.

Successful completion (with the attendant risk of failure) of such a paradoxical task appears to require a clear description from the outset of the T-C relationship as a *working relationship*, a *functional relationship*, and putting emphasis on what happens outside rather than inside the therapy room.

It should finally be noted that this conditioning perspective was not formulated on the basis of research. It is somewhat surprising that an approach that so heavily emphasises the influence of the environment has performed so little research concerning this T-C relationship. A therapist is after all the most important stimulus in a therapy context!

Useful research could take different directions. We already mentioned a number of research strategies that relate to the therapy process in itself, a within-session analysis of the therapist-client interaction. The conditioning framework can help to define the functional units.

We believe it would be similarly useful, and perhaps conceptually clearer, to perform research that uses existing experimental paradigms that have both a methodological and theoretical foundation as a starting point for a study of the T-C relationship. Consider the following examples.

Experimental social psychology includes the well-known paradigm of “social facilitation” (for a comprehensive literature overview, see Guerin, 1986). How does the presence of a socius influence a participant’s behaviour? The presence of a socius sometimes appears to facilitate or inhibit completion of a task. The latter effect is then ascribed to an increase in tension that especially hampers the execution of more complex tasks. Using the existing experimental literature and theories, every creative researcher can now formulate a specific research question focused on the influence of a therapist’s presence on a client’s execution of a task. In accordance with the above-mentioned description of a therapist as an inhibitory stimulus, it is to be expected that his presence will not have a disruptive effect on a client’s execution of a task.

Alternatively, the well-known “priming” paradigm from cognitive psychology might be used. Presenting a stimulus activates an associative network that causes stimuli that are related in meaning, to subsequently be processed more easily than unrelated stimuli. This again creates a series of possibilities to find out in which meaning pattern a client situates his therapist (and perhaps vice versa), as well as how this meaning pattern evolves as a function of therapy progress (for a broader application of the priming paradigm, see Bower, 1986).

What connects these suggestions is their mutual attempt to identify a therapist’s meaning to his client in a scientifically sound manner, and this in a more indirect manner than through the use of questionnaires. Such research may strike a veteran clinician as artificial. Research, however, does not have as its purpose to simulate “reality” as faithfully as possible, but instead aims to critically question our vocabulary of concepts, and such critical questioning should ideally take place in a controlled experimental setting.

## Conclusion

The “labels” dividing the different psychotherapy models will no doubt cease to exist at some point. For now, however, we do not think it realistic this will happen in the near future. As long as these oppositions purely spring from a difference in the terms used, this does not pose any problems and instead is purely a matter of translating, with the precise choice of language guided by the therapist’s aesthetic preference. But these differences are likely of a more elemental nature and interweaved with the fundamental discussion about the core fundaments of our basic discipline: psychology.

## Footnotes

This paper refers to the therapist with male pronouns. Because the client is also continually presented as male, any type of male chauvinism is avoided.

Perhaps not coincidentally, in this study the computer was given the name ‘DAD’ (device for automated desensitisation).

We use a broad UCS definition here, which helps us to circumvent the question of whether this stimulus even has an inherent well-defined meaning (*unconditional* in the true sense of the word) or whether it flows from a prior learning process.

It here becomes clear that behaviour therapy does take into consideration a client’s feelings – not as a goal in and of itself, but with the aim of discovering which stimulus operations might fuel these feelings (functional analysis).

One of the first ever attempts to “translate” psychoanalytic concepts into learning psychology is ascribed to Dollard and Miller (1950). They saw transfer more as a form of stimulus generalization.
